# Influence of Scuba Diving on the Quality of Life of People with Physical Disabilities

**DOI:** 10.3390/healthcare10050761

**Published:** 2022-04-20

**Authors:** Gabriela Henrykowska, Joanna Soin, Katarzyna Pleskacz, Piotr Siermontowski

**Affiliations:** 1 Department of Epidemiology and Public Health, Medical University of Lodz, 90-752 Lodz, Poland; 2 Institute of Health Sciences, Stefan Batory State University, 96-100 Skierniewice, Poland; jsoin@pusb.pl; 3 Faculty of Physics, Astronomy and Informatics, Institute of Physics, Nicolaus Copernicus University in Toruń, 87-100 Toruń, Poland; pleskacz@doktorant.umk.pl; 4 Department of Underwater Work Technology, Polish Naval Academy of the Heroes of Westerplatte, 81-103 Gdynia, Poland; nurdok@o2.pl

**Keywords:** scuba diving, rehabilitation in disability, quality of life

## Abstract

The aim of the study was to assess quality of life related to mental and physical health among divers and non-divers with physical disabilities. The examined group consisted of 240 disabled people (both genders). The SF-36 questionnaire (Short-Form Health Survey) was used to measure the overall sense of health-related quality of life. Moreover, the authors’ survey was also used in the study. There was a significant difference (*p* < 0.05) in the self-assessment of the quality of life (physical functioning, social functioning, mental health, and vitality) between the examined diving and non-diving groups. In other areas evaluated with the use of the SF-36 questionnaire, i.e., limitation in performing roles due to emotional problems and pain, limitations in performing roles due to physical health, a tendency to a higher rating was noticed in the group of divers. Scuba diving can improve various components of the life-quality of people with disabilities, and in general can be seen as a form of physical activity and rehabilitation for people with disabilities. However, it is necessary to conduct extensive research in this area.

## 1. Introduction

Disability is an issue that is gaining more and more importance for the functioning of modern societies. The share of people with disabilities in the global population is continuing to increase. It is estimated that 4–7 million people with disabilities currently live in Poland, among whom over 3 million have disability certificates issued by an appropriate authority [1]. The spectrum of effects of disability is extremely broad. They concern not only the physical domain, but also the mental and social dimension and can significantly affect the quality of life.

Disabilities can be divided into mental and physical disabilities. The first consist in a significantly reduced general level of intellectual performance and deficits in adaptive behavior. The second group includes impairments of the locomotor system, e.g., lack of limbs or parts of them (limbs not formed in foetal life or amputated as a result of illness or accident), impairments of the nervous system (e.g., paraplegia, tetraplegia), incorrect formation of the skeleton, joint impairments (e.g., caused by rheumatic diseases, sports). In addition, it includes sensory dysfunctions, such as visual, hearing and speech impairments and chronic diseases of the internal organs.

The quality of life is described very differently and there is no single definition of the concept. The World Health Organization (WHO) defines the quality of life (QoL) as: “An individual’s perception of their position in life in the context of the culture in which they live and in relation to their goals, expectations, standards and concerns” [2]. It allows to consider all issues containing the physical, mental, and sociological domains.

The term health-related quality of life (HRQOL) “reflects the impact of perceived health on an individual’s ability to live a fulfilling life” [3]. This concept of the quality of life includes the ability to perform physical, mental, and social functions in relation to the limitations that may be caused by a particular disease or disability [4].

Proper understanding of the term “quality of life” is important in minimizing the effects of a disability on life quality. The evaluation of HRQOL components can lead to the modification and/or improvement of rehabilitation with many benefits for people with disabilities.

Disability influences the perception of quality of life in a varied and multifaceted way. The quality of life of people with disabilities depends on many factors. Socio-demographic factors include: place of residence, educational background, duration of disability, age or marital status. Other important factors include: presence or absence of architectural and technical barriers, presence of devices facilitating communication, adequate financial resources, having or not having employment, quality of contacts and relations with family and other close people, possibility to develop one’s interests and carry out physical activity.

Many studies have demonstrated that various forms of physical activity have a significant impact on improving the quality of life of people with physical disabilities [5,6,7]. Moreover, regular physical activity is an effective factor that plays an important role in prevention and rehabilitation. It prevents secondary complications that may arise from physical disability [7].

In recent years, self-contained underwater breathing apparatus (SCUBA) diving has gained the popularity and prestige and has acquired the image of an interesting leisure time activity. Interest in it is exhibited by both healthy and disabled people. Contrary to snorkeling (swimming near the water’s surface with the use of a mask and a breathing tube), scuba diving allows divers to breathe comfortably while being underwater, completely immersing themselves in the marine depths. Diving can be used as a comprehensive rehabilitation method. This type of therapy makes it possible to improve both the physical and mental domain of human life [8,9,10].

The aquatic environment extends the possibilities for people with disabilities. Exercises in water, for which one needs to learn how to breathe properly, have a stimulating effect on the respiratory and circulatory systems [6]. Water facilitates the performance of movements that could be significantly limited in the land environment. The aquatic environment helps in relaxing contracted muscles and increases the range of motion. It gives people with disabilities the possibility to sense the position and movement of their bodies and limbs, to give direction to movements and increase the sense of independence [9,11,12].

Diving may help to overcome limitations caused by disability [9]; it often happens that diving enables disabled people to perform better than their able-bodied peers who declare average levels of physical activity. To a person with disability, doing something unusual makes her/him feel special, which clearly facilitates coming to terms with one’s status [11,13]. Scuba diving could increase the engagement in physical activity of people with disabilities, by which it extends their participation in social life and opens them up to new experiences. It could reduce the level of anxiety and depression, improve self-esteem and self-confidence [5,8,9,10,13]. Nevertheless, one may not forget potential risks and hazards linked to scuba diving. Factors that impact human body, apart from the initial health condition, are: pressure under water, breathing gas, temperature, type of the diving environment, mental barriers and possible problems with the equipment [14,15].

People with disabilities are permanently looking for rehabilitation forms and methods that could contribute to improving their performance in everyday life [16]. Considering the fact that scuba diving is emerging as a fashionable and very interesting leisure time activity, we should find out whether it really produces therapeutic outcomes and is safe for people with disabilities.

The aim of the study was to compare the quality of life of disabled divers and people with disabilities who have not had the opportunity to dive yet. (This is one of the initial stages of an international research project on the benefits of scuba diving, as well as the difficulties and fears experienced by people with disabilities.)

## 2. Materials and Methods

### 2.1. Methods

In the non-experimental cross-sectional study, we used as a research tool the questionnaires SF-36 and SF-12 (version 1.0) [17] intended to measure the quality of life, and the authors’ original questionnaire providing disability information, socio-demographic and anthropometric data (body mass and weight), which contained closed, semi-open, and an open set of questions created using Google Forms.

The SF-36 questionnaire is a tool for measuring quality of life that can be used for the general population and people with various health problems. It consists of 36 items that add up to two summary scores. The Physical Composite Summary Score (PCS) includes 4 dimensions: physical functioning (PF), role limitations due to physical problems (RP), bodily pain (BP), and general health (GH). The Mental Composite Summary Score (MCS) includes: vitality (VT), social functioning (SF), role limitations due to emotional problems (RE) and mental health (MH). Scores range between 0 and 100; the higher the score, the better the health-related quality of life.

Interviewed persons were asked about the type and degree of disability and the type of regularly practiced physical activities. No more detailed data were collected concerning their health status as, according to Polish regulations, a person who wants to practice diving is not required to hold a health certificate confirming her/his condition (including her/his lung functions). Pursuant to Polish law, in recreational diving, no prior health check or classification of health status are required. It is enough if a potential scuba diver declares that he/she wants to engage in this activity and he/she believes that her/his health permits it [18]. 

All survey respondents who dived declared being active divers who perform the activity on a regular basis. Information about the number and frequency of diving and the period of time since the last diving was not gathered for this study. We only wanted to determine whether the respondent did not dive at all or declared to be a regular diver.

Questionnaire forms (all forms making up one survey) were distributed online, using one of the social media sites.

**Figure 1:**
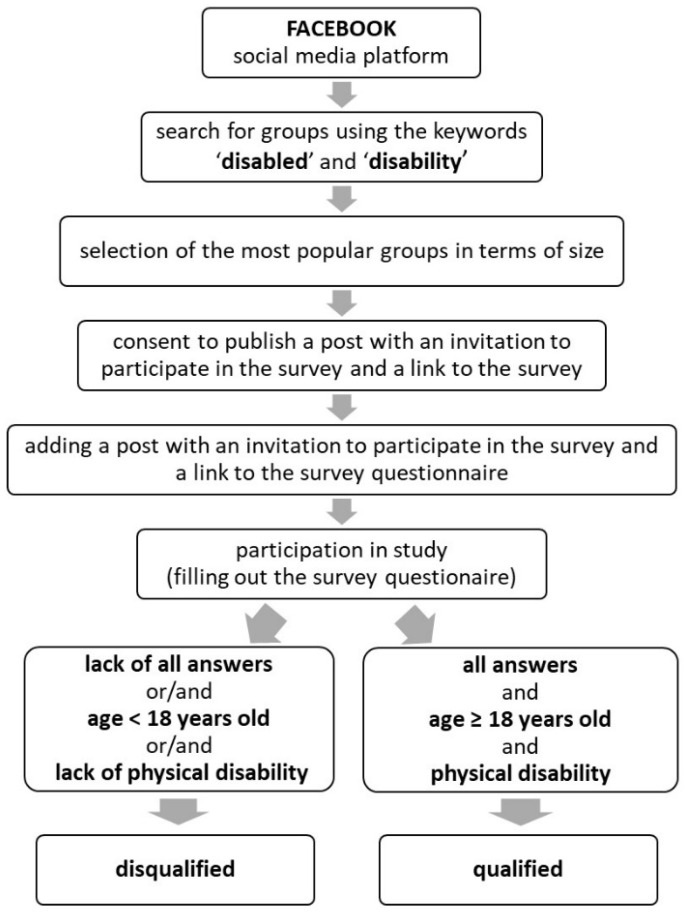
Infographic that describes the organizational structure of the survey.

The research covered exclusively persons with physical disabilities, who are members of thematic groups focused on disabled persons and their engagement in sports activities on one of social media platforms (Facebook). The most popular groups were selected in terms of the number of associated users using the keywords: ‘disabled’, ‘disability’. At the time of selection, the groups were made up of between several to tens of thousands of users (Figure 1).

Upon prior consent of administrators of individual thematic groups, we posted an invitation to take part in the survey and a survey link. 

When it comes to participation in the study, the limitations concerned age (as the participants were supposed to be 18+) and disability (those reporting no physical disability were excluded). 

In further analyses we focused only on questionnaires meeting the above criteria and which were fully completed by respondents. Participation in the study was voluntary. Respondents (who remained anonymous throughout the whole process) could stop or withdraw from the filling out of the questionnaire at a time of their choice. Even though researchers strictly observed data confidentiality principles, a reservation was made concerning the risk of the confidentiality of data being infringed when they are transferred over the Internet (as that lies beyond the control of the survey team). A clear statement was also included that the filling out of the questionnaire is equivalent to giving one’s consent to participate in the study.

Data were analyzed by Shapiro–Wilk test, U Mann–Whitney test, ANOVA, Tukey’s post hoc test and a descriptive statistical analysis

Data analysis began with the examination of the distribution of dependent variables that can be found in Table 1. For this purpose, the Shapiro–Wilk test was carried out. Based on this test, we found out that the examined variables are not normally distributed which is why non-parametric tests should be used for further analysis. The significance level was taken as *p* < 0.05. 

The Mann–Whitney U test was used for making comparisons between groups A and B. The results of this analysis are presented in Table 2, which includes mean values, standard deviation, confidence interval (95% Confidence Interval (95% CI)), and *p*-value. In addition, the effect size and its categorization using Cohen’s scale were provided to determine the practical significance of the effects observed in the study. For the sake of clarity, the following explanations have been added: depending on the range, the effect size was interpreted as d < 0.2–negligible, 0.2 ≤ d < 0.5–small, 0.5 ≤ d < 0.8–medium, and d ≥ 0.8–large. 

The independent variable that informs about the degree of disability describes three states: mild, moderate, and severe. A division of this type is proper for the examination of the differences between groups for each degree of disability for the whole studied group. 

To carry out such examination, Tukey’s post hoc test and a descriptive statistical analysis were used.

Statistics calculations were conducted using the SigmaPlot (Systat Software, San Jose, CA, USA).

### 2.2. Study Group

The questionnaire was addressed to people with disabilities, who are members of thematic groups focused on disabled persons and their engagement in sports activities on one of social media platforms (Facebook). 

The respondents were divided into two groups. Group A was composed of people who (before the study) had never participated in diving classes (*n* = 118). Group B included people with disabilities who already had experienced diving (*n* = 64).

Before becoming disabled (as a result of illness or injury), no respondents had diving experience. People from Group B (divers) declared that diving was physical activity that they practiced regularly. However, information about the amount and frequency of diving and the period of time since the last dive was not gathered for this study. We only wanted to determine whether the respondent had no diving experience at all or declared themselves to be a regular diver.

The survey was conducted among 240 respondents (sex: male and female) with physical disability. Correctly filled out questionnaires were submitted by 64 disabled persons practicing scuba diving and 118 disabled persons who had no previous experience in diving. Answers provided by 58 respondents were rejected for formal reasons (mistakes made when filling the form or lack of responses). Further analyses were carried out on the basis of 182 correctly completed questionnaires (group A: *n* = 118; group B: *n* = 64). The difference in group size was acceptable and did not affect the results [19].

Pursuant to the Polish regulations, the health status of a given individual is defined by the authority responsible for assessing degrees of disability and classifying disabilities into one of the three below described categories: severe, moderate, and mild [1].

In accordance with the regulations, a person with severe disability is someone who suffers from an impaired ability of her/his organism, cannot work, or can work only in a protected working environment. Being unable to lead an independent life, such a person requires permanent or long-term assistance from other people in order to fulfil her/his social roles. 

Moderate disability is assessed for persons with physical impairment who are unable to work or able to work only in a protected working environment. A person with moderate disability may also require temporary or partial assistance from others to be able to fulfil her/his social roles. 

Finally, a person with a mild degree of disability is someone with a physical impairment of the body that significantly limits her/his ability to perform work in comparison with the ability exhibited by a person having similar occupational skills and full psychological and physical capacity. It may also be a person whose limitations in fulfilling social roles can be compensated through the use of equipment such as orthopedic appliances, aids or technical devices [1].

The study was approved by the Committee of Research Ethics operating at the Naval Academy of the Heroes of Westerplatte (Decision of Ethic Committee #4/21).

This research is the first part and the first step in an international survey on benefits, difficulties, and fears that scuba diving may produce in people with disabilities.

## 3. Results

The age of the respondents ranged between 22 and 75 years. The disability of 28.02% of the respondents was congenital. A total of 39% of respondents became disabled as a result of a disease; for 32.8%, disability was the result of an injury. In the examined group, dysfunctions of the musculoskeletal system were the reasons for disability in 78% respondents (142 people); 4.4% of the respondents stated that their disability concerned vision dysfunction, and the remaining respondents suffered from neurological diseases. 

A total of 48.9% of the respondents held a certificate of disability stating that their condition was severe; 32.4% of respondents were diagnosed with moderate and 18.7% with mild degree of disability. Among the individuals with acquired disability, 31.3% of respondents had lived with disability for over 10 years. The largest group (49.5%) was made up of people who had been disabled for 6 to 10 years. A total of 19.2% of respondents had been disabled for between a year and 5 years. 

A total of 108 people (59.3% of the respondents) had the opportunity to take part in rehabilitation classes conducted in an aquatic environment; 64 individuals, i.e., 35.17% of all respondents, had taken part in scuba-diving classes. An extensive description of the examined group is presented in Table 1 [16].

Average age was rather similar in both groups (Group A, Non-divers: 36.51 ± 12.63; Group B, divers: 38.82 ± 9.79); no statistically significant differences between them were revealed (*p* > 0.05). Additionally, when analyzing the age of the researched individuals in the context of the degree of their disability, no statistically relevant differences were identified between the groups. 

Independent of the degree of disability, forms of physical activity practiced by the respondents (from Groups A and B) were very similar. The only difference was their active involvement (or not) in diving. Respondents could identify/list types of physical activity that they regularly practice. Group A (“non-divers”) were engaged in activities such as: cycling/handbike, amputee football, swimming, volleyball, wheelchair basketball, bowling, boccia, horse riding, and gymnastics as part of their rehab. Apart from diving, Group B (“divers”) listed swimming, bike/handbike, AMP football, volleyball, wheelchair basketball, bowling, as well as boccia, and climbing. 

One third of the Group A respondents declared they did not practice any physical activity. Among them, 53% were people with mild disability, 34.6% people with moderate disability, and only 9.6% people with severe disability.

Health perceptions of the SF-36 domains varied. In all scales of the SF-36, the scoring of all domains was lower in non-divers as compared to divers. Statistically significant differences were observed (*p* < 0.05) in four domains: physical functioning, vitality, social functioning, and mental health (Table 2).

To more thoroughly examine differences between the groups, we used multivariant analysis and post hoc Tukey’s test for unequal sample sizes. The assumed significance level was *p* < 0.05 (Table 3).

Independent of the degree of disability, statistically higher quality of life (*p* < 0.05) was observed for people who practiced diving in terms of both mental health and functioning in everyday life situations. Statistically significantly higher quality of life was also reported for the group of divers with severe and mild disabilities in the vitality domain. In addition, statistically significant differences were observed in general health, physical functioning, and role limitations due to emotional problems. No statistically significant differences were found between people with disabilities practicing scuba diving and non-divers with disabilities only in the area of role limitations due to physical problems and bodily pain (Table 3). 

Due to the small size of the cohort and the fact that it is made up of disabled divers, we declined/were unwilling to compare the SF36 domains with existing age- and gender-specific reference values and to estimate the position of the cohort in terms of QoL.

The health status of the analyzed population, including the mental (MCS) and physical component summary scale (PCS), is reported in Table 4. The mean PCS score obtained from group A (48.96) was significantly lower than the score obtained from group B (56.56). The mean MCS score was also significantly lower in the non-divers group (62.81 vs. 79.19). 

## 4. Discussion

There is only a handful of available studies describing divers with disabilities. To date, researchers have not attempted to describe the quality of life of divers with disabilities in a way similar to that proposed in our study. Therefore, this discussion is not conducted in a classical way.

When analyzing the available references or investigating the proposed treatment methods for various diseases, various studies were found describing the quality of life of specific populations [20,21,22,23]. Unfortunately, the number of reports describing the quality of life of divers with disabilities is limited. This may be due to the fact that wider interest in scuba diving in the disabled community and in the world of science has increased only in recent years [16]. Previous studies of the impact of scuba diving on the health and quality of life of disabled people were conducted on limited groups of respondents [10,11,13,24]. Our study included only 64 divers with disabilities, but this was still the biggest group of scuba divers with disabilities analyzed ever. 

Williamson et al. [24] reported that diving can impact the quality of life of people with disabilities by improving their physical and mental condition. Our survey demonstrated that those engaged in diving declared better quality of life with regard to mental and physical health compared to non-divers. These results can be explained by and attributed to the specific qualities of the water environment, which gives significant support to people with disabilities. Scuba diving provides the feeling of lightness and freedom from illness or disability [11]. After immersion into water, the loss of body weight enables and facilitates movements that are impossible or very difficult on land [9,12]. 

According to some studies, the aquatic environment reduces pain and improves physical functions in people with lower back pain [25]. Aquatic exercises improve the flexibility, mobility, and strength in patients with osteoarthritis [26]. Dundar et al. [22] showed that exercises in water improve the quality of life measured in six dimensions of the SF-36—bodily pain, general health, vitality, social functioning, role limitations due to emotional problems, and general mental health—among patients with ankylosing spondylitis. Aidar [21] also confirmed the influence of exercises in water on the improvement of life quality of disabled people with ischemic vascular incidents. Significant improvement in both mental and physical components assessed by the SF-36 questionnaire was observed. In our research we observed a significantly higher score for both physical and mental components of the questionnaire among diving respondents with disabilities. These results cannot be attributed solely to the structure of the groups viewed through the levels of disability. According to the Polish regulations, each level of disability covers various dysfunctions that in fact cannot be compared (e.g., visual impairment vs. arm amputation). Certainly, with increased group sizes (especially Group B), it would be to carry out analyses by comparing groups broken down into types of disability.

Aquatic exercises and scuba diving increase the mobility of the respiratory system. McNamara reported the improvement of the peak and endurance exercise capacity in people with chronic obstructive pulmonary disease after the breathing training in water [6]. In our study, the diving respondents also gave statistically significantly higher scores to the level of HRQoL in general health and physical functioning categories. In the present study, no statistically significant difference was observed between the analyzed groups with respect to the category of role limitations caused by emotional problems. Studies conducted by other authors do not provide analyses that could be used as a reference for our results.

Perez-de la Cruz [23] provided evidence that Ai Chi aquatic therapy programs increase the quality of life of people suffering from Parkinson’s disease; they reduce body pain and depression. In our study, no statistically significant difference was found between respondent groups in the body pain dimension. However, we observed lower scores in this HRQoL parameter in the non-divers group.

Diving as a recreational activity can offer many health benefits, such as reduction of perceived stress and fatigue, and improvement of well-being and self-acceptance [10,11,25]. The positive effect of scuba diving on the increase of the well-being was also evidenced in the present study: the level of vitality was significantly higher in the group of diving respondents.

Cheng and Diamond [8] pointed out that diving affects the quality of life by improving self-esteem and reducing depression. In our research, we also observed a significantly higher scoring of the mental health (MH) category in the group of divers with disabilities.

Morgan et al. [10] indicated that scuba diving could help to decrease anxiety and depression levels; it may improve social functioning and relationships (with the social environment). Aganović’s research also showed an improvement in the self-esteem and social functioning of people after amputation [13]. Carin-Levy and Jones [11] indicated improved self-consciousness and enhanced social experiences in people with disabilities who practiced diving. In our study, it was also divers also scored significantly higher on the social functioning scale. There was no statistically significant difference in the emotional role category between respondent groups A and B. However, scores for this parameter given by divers were slightly higher than those scored by non-divers.

One needs to bear in mind, however, that scuba diving does entail various risks. Before starting the adventure with this form of physical activity, a physician specializing in underwater medicine or diving and hyperbaric medicine should carry out a careful examination of a candidate’s health. Diving practice should be preceded with specialist training and necessitates specialist equipment (adjusted individually to each disabled person) to avoid serious injuries or death [14,24]. Everyone who dives must be aware of dangers and risks that might result from health status but also from factors that influence a diver and are triggered by the change of environment (e.g., underwater pressure, respiratory gases, temperature) [15,27,28]. A potential diver must also be aware of the consequences of decompression sickness, air-gas embolism, oxygen toxicity, nitrogen narcosis, or simply the risk of drowning [29]. Other problems may also arise, such as psychological barriers like claustrophobia or problems related to the specificity of diving equipment [14,30]. These hazards entail the risk of disability worsening.

To reduce risks involved in scuba diving to a minimum, a person’s disability cannot prevent her/him from independently learning to dive or dive with the help of assistants. Moreover, water reservoirs for scuba diving should be selected carefully so as not to generate additional problems to disabled persons. For instance, people with paraplegia should not dive in rivers, as they have limited abilities to cope with the stream [14,15]. 

For those who are interested in it, however, it could become an alternative form of rehabilitation. Diving could be an excellent example of how to eliminate barriers and achieve goals often unattainable to individuals without disabilities [14,16].

Results of various studies, as well as results of our own analysis, suggest that diving could be an important factor in improving the life quality of people with disabilities. 

Given the growing interest in scuba diving exhibited by people with disabilities, further studies are needed that would ensure practicing it in the safest way possible and offer full awareness of risks and their disability-related limitations

## 5. Conclusions

The quality of life of diving individuals (independently of the degree of their disability) was higher with regard to mental health and social performance compared to the non-diving group.

The fact that we observed a statistically significant difference in the perception of health-related QoL aspects between divers and non-divers allows us to assume that scuba diving can improve various components of the quality of life of people with disabilities.

To people with disabilities wishing to engage in physical exercises, diving can be a way to improve their quality of life. However, prior to practicing scuba diving, they must undergo a thorough medical assessment performed by a diving medicine specialist and be informed about health-related risks involved in this activity and potential worsening of their disability. 

## Figures and Tables

**Table 1:** Description of the analyzed group [16].

Category	Non-Divers (*n* = 118)	Divers (*n* = 64)
	Percent	Percent
Men	61.8	73.4
women	38.2	26.6
severe degree of disability	56.6	42.2
moderate degree of disability	22	12.5
mild degree of disability	25.4	45.3
congenital disability	27.97	28.12
disabled as a result of a disease	51.70	15.63
disabled as a result of an injury	20.33	56.25
disability duration: 1–5 years	23.73	10.94
disability duration: 6–10 years	39.83	67.19
disability duration: more than 10 years	36.44	21.87
musculoskeletal system dysfunctions	75.42	82.81
dysfunctions caused by neurological diseases	18.65	10.93
vision dysfunction	5.08	3.12
hearing impairment	0.85	3.12

**Table 2:** Scores in eight domains of SF-36 provided by diving and non-diving people with disabilities.

SF-36 Domains	Non-Divers (A)					Divers (B)					*p*	Effect Size	Categorized Effect Sizes
	Mean	SD	95% Cl			Mean	SD	95% Cl					
Physical functioning	27.38	11.21	22.65	-	31.92	40.9	22.32	32.33	-	49.39	0.006	1.21	large
Role limitation (physical)	58.36	19.59	56.10	-	67.20	65.49	29.54	58.20	-	73.05	0.381	0.36	
Bodily pain	55.75	25.95	68.58	-	76.62	58.80	26.10	69.18	-	79.77	0.603	0.12	
General health	54.33	21.73	49.62	-	54.87	61.07	21.97	66.62	-	74.63	0.673	0.31	
Social functioning	61.80	26.50	64.60	-	68.48	87.30	18.50	82.07	-	87.43	0.003	0.96	large
Role limitation (emotional)	70.94	26.62	48.01	-	56.99	74.22	21.80	47.77	-	60.90	0.640	0.12	
Mental health	66.43	10.60	57.48	-	64.56	84.80	13.70	82.80	-	91.42	0.012	0.73	medium
Vitality	52.08	13.01	51.81	-	57.10	70.46	17.60	57.55	-	64.65	0.009	0.41	medium

**Table 3:** Comparison of SF-36 domains between diving and non-diving people with disabilities depend on degrees of disability (post hoc Tukey test).

Degree of Disability	SF-36 Domains	Non-Divers (A)		Divers (B)		*p*	Effect Size	Categorized Effect Sizes
		Mean	SD	Mean	SD			
**severe**	Physical functioning	16.69	22.85	17.78	23.79	0.864	0.05	
	Role limitations physical	42.74	29.82	43.52	29.90	0.924	0.03	
	Role limitations emotional	72.04	21.92	72.84	22.72	0.895	0.04	
	Vitality	50.16	14.82	66.11	16.89	0.000	1.08	large
	Mental health	66.26	8.92	84.44	9.76	0.000	2.04	large
	Bodily pain	46.85	17.30	46.02	17.56	0.860	−0.05	
	Social functioning	56.85	21.31	77.78	20.02	0.001	0.98	large
	General health	48.23	11.88	53.15	13.53	0.148	0.41	
**moderate**	Physical functioning	40.77	25.44	51.25	35.93	0.461	0.41	
	Role limitations physical	86.54	12.71	78.13	8.84	0.170	−0.66	
	Role limitations emotional	80.77	16.79	62.50	27.82	0.074	−1.09	
	Vitality	56.54	13.84	62.50	10.00	0.370	0.43	
	Mental Health	70.00	10.87	88.50	5.83	0.001	1.70	large
	Bodily pain	66.35	24.80	57.19	36.95	0.517	−0.37	
	Social functioning	70.19	13.73	93.75	11.57	0.001	1.72	large
	General health	65.79	11.54	60.63	15.45	0.415	−0.45	
**mild**	Physical functioning	37.50	20.96	59.48	29.53	0.002	1.05	large
	Role limitations physical	79.17	9.48	82.76	17.81	0.339	0.38	
	Role limitations emotional	66.67	24.76	79.31	16.46	0.026	0.51	medium
	Vitality	52.83	13.50	77.07	14.42	0.000	1.80	large
	Mental health	64.13	13.10	84.00	12.51	0.000	1.52	large
	Bodily pain	52.17	32.36	61.29	28.43	0.259	0.28	
	Social functioning	61.67	17.04	93.97	10.89	0.000	1.90	large
	General health	57.50	15.13	68.64	10.24	0.002	0.74	medium

**Table 4:** Health status assessment of diving and non-diving people with disabilities.

SF-36 Summary Scales	Non-Divers (A)		Divers (B)		*p*-Value
	Mean	SD	Mean	SD	
PCS	48.96	19.6	56.56	24.9	0.001 *
MCS	62.81	21.4	79.19	19.9	0.032 *

* *p* < 0.05.

## Data Availability

Not applicable.
